# Preoperative Mapping of the Sensorimotor Cortex: Comparative Assessment of Task-Based and Resting-State fMRI

**DOI:** 10.1371/journal.pone.0098860

**Published:** 2014-06-10

**Authors:** Cristina Rosazza, Domenico Aquino, Ludovico D’Incerti, Roberto Cordella, Adrian Andronache, Domenico Zacà, Maria Grazia Bruzzone, Giovanni Tringali, Ludovico Minati

**Affiliations:** 1 Neuroradiology Department, Fondazione IRCCS Istituto Neurologico “Carlo Besta”, Milano, Italy; 2 Scientific Department, Fondazione IRCCS Istituto Neurologico “Carlo Besta”, Milano, Italy; 3 Neurosurgery Department, Fondazione IRCCS Istituto Neurologico “Carlo Besta”, Milano, Italy; 4 Center for Mind/Brain Sciences (CIMeC), University of Trento, Trento, Italy; Max Planck Institute for Human Cognitive and Brain Sciences, Germany

## Abstract

Resting state fMRI (rs-fMRI) has recently been considered as a possible complement or alternative to task-based fMRI (tb-fMRI) for presurgical mapping. However, evidence of its usefulness remains scant, because existing studies have investigated relatively small samples and focused primarily on qualitative evaluation. The aim of this study is to investigate the clinical usefulness of rs-fMRI in the context of presurgical mapping of motor functions, and in particular to determine the degree of correspondence with tb-fMRI which, while not a gold-standard, is commonly used in preoperative setting. A group of 13 patients with lesions close to the sensorimotor cortex underwent rs-fMRI and tb-fMRI to localize the hand, foot and mouth motor areas. We assessed quantitatively the degree of correspondence between multiple rs-fMRI analyses (independent-component and seed-based analyses) and tb-fMRI, with reference to sensitivity and specificity of rs-fMRI with respect to tb-fMRI, and centre-of-mass distances. Agreement with electro-cortical stimulation (ECS) was also investigated, and a traditional map thresholding approach based on agreement between two experienced operators was compared to an automatic threshold determination method. Rs-fMRI can localize the sensorimotor cortex successfully, providing anatomical specificity for hand, foot and mouth motor subregions, in particular with seed-based analyses. Agreement with tb-fMRI was only partial and rs-fMRI tended to provide larger patterns of correlated activity. With respect to the ECS data available, rs-fMRI and tb-fMRI performed comparably, even though the shortest distance to stimulation points was observed for the latter. Notably, the results of both were on the whole robust to thresholding procedure. Localization performed by rs-fMRI is not equivalent to tb-fMRI, hence rs-fMRI cannot be considered as an outright replacement for tb-fMRI. Nevertheless, since there is significant agreement between the two techniques, rs-fMRI can be considered with caution as a potential alternative to tb-fMRI when patients are unable to perform the task.

## Introduction

Accurate localization of functionally-relevant brain areas is important for presurgical planning, as it helps optimize resection and minimize postoperative neurological deficits. Electro-cortical stimulation (ECS) is the gold standard procedure for mapping brain function intraoperatively; while it is generally very reliable, it has disadvantages in that it cannot be used at the presurgical planning stage but only intra-operatively, it is limited in access by the surgical operculum, and may occasionally yield inconclusive results due to anesthesiological issues [1]–[5].

In virtue of its non-invasiveness and widespread availability, functional MRI (fMRI) has gained increasing acceptance over the last two decades as an important tool for presurgical planning, in particular in cases where Rolandic landmarks cannot be clearly identified on morphological images [6], [7]. Depending on lesion localization, typical protocols include foot, hand and mouth motor tasks for localizing the paracentral lobule, precentral knob and inferior peri-Sylvian motor area, respectively [8]. While it does not represent a gold standard of activity localization, particularly due to the potential effects of neurovascular confounds, task-based fMRI (tb-fMRI) has good agreement with intraoperative ECS mapping [4], [7], [9]–[13] and enables rapid assessment of the distance between the motor cortex and the lesion [14].

However, tb-fMRI can fail in patients who cannot perform the tasks satisfactorily because of neurological deficits or neurocognitive state. In such cases, a possible alternative is localizing the motor cortex through its spontaneous activity. This is a more recent approach which relies on the fact that spontaneous fluctuations in the blood oxygen level-related (BOLD) signal are temporally-coherent within discrete networks, that appear to correspond to specific brain circuits involved in motor control, vision and cognitive integration [15]. In their seminal study, Biswal et al. [16] demonstrated that under wakeful rest the BOLD signal fluctuations of the left and right sensorimotor cortex and supplementary motor area are correlated, leading to the notion of a sensorimotor resting-state network. They additionally showed that spontaneous and task-based activity maps are strikingly similar in topographical localization. Further work on healthy controls has demonstrated that topographical maps obtained via resting-state fMRI (rs-fMRI) are highly consistent and reproducible across subjects and sessions [17]–[19], and that tb-fMRI and rs-fMRI yield moderately consistent results in healthy controls [20]–[22].

Rs-fMRI has been considered as a means of presurgical planning in a number of exploratory studies, which confirmed its potential usefulness [23]–[27]. In particular, Zhang et al. [23] studied four patients with tumors infiltrating the sensory and motor cortices, comparing rs-fMRI with tb-fMRI and ECS. Liu et al. [24] and Kokkonen et al. [25] conducted similar evaluations respectively on 6 and 8 patients with lesions neighboring the motor cortex. These studies used different resting-state data analysis techniques. In fact, multiple approaches are available, the most common ones being independent component analysis (ICA, based on the extraction of reference time courses from independent distributed cortical networks) and seed-based analysis (SBA, based on the extraction of reference time courses by averaging over pre-specified regions), with regions-of-interest (ROIs) defined from structural or functional anatomy on the healthy hemisphere. While ICA and SBA provide correlated results, the correspondence is only partial and it remains unclear which approach is preferable for presurgical applications of motor rs-fMRI [15], [17], [28]–[30].

Evidence available to date suggests that rs-fMRI may be useful in presurgical planning, but there are limitations related to methodological differences, small sample size and the fact that most of the studies considered a single tb-fMRI task as reference. To contribute to defining the potential usefulness of rs-fMRI in presurgical planning of motor function, we report a comparison of rs-fMRI with tb-fMRI and ECS in a sample of 13 patients with etiologically heterogeneous lesions in the sensorimotor area. While tb-fMRI is not a gold standard of activation localization, its widespread clinical use warrants its consideration as a reference for determining the potential usefulness of rs-fMRI in preoperative setting. Half of the patients performed more than one active task and we conducted a comprehensive comparison of rs-fMRI obtained with ICA and SBA methods, with a focus on quantitative rather than visual comparison.

## Methods

### Patients

The study was approved by the institutional review board (code: fMRI-riposo) of Fondazione IRCCS Istituto Neurologico Carlo Besta. A total of 13 presurgical patients (3 female, age range 31–74, median 52) necessitating resection of a lesion neighboring the motor cortex were recruited after providing written informed consent. Preoperative function and postoperative outcome at 1 week and 3 months were assessed using the British Medical Research Council Scale (BMRC) for muscle strength. Lesion localization, pathology and volume are reported in Table 1.

**Table 1:** Patient clinical and demographic data.

Case	Age	Sex	Lesion localization	Pathology	Field strength	Lesion volume/ml	BMRC^a^ score		
							Pre-op	1 week post-op	3 months post-op
1	48	M	R motor	Focal cortical displasia	1.5 T	85	5	5	5
2	34	F	R premotor	Low grade glioma	1.5 T	250	5	5	5
3	59	F	R motor	Glioblastoma multiforme	1.5 T	144	2	1	2
4	51	M	R premotor	Low grade glioma	1.5 T	58	5	5	5
5	52	M	R premotor (fronto-opercolar)	Cavernous angioma	3.0 T	25	5	5	5
6	57	M	L premotor, motor (and plegic left hand from birth)	Lung metastasis	3.0 T	35	1	1	1
7	47	M	L somatosensory	Focal cortical displasia	3.0 T	182	5	5	5
8	62	M	R motor	Glioblastoma multiforme	3.0 T	394	5	5	5
9	31	M	L somatosensory, motor	High grade glioma	3.0 T	638	4	4	4
10	35	M	R somatosensory, motor	Lung metastasis	3.0 T	41	4	4	4
11	56	M	L motor, somatosensory	Lymphoma	3.0 T	86	4	4	4
12	68	M	L motor, somatosensory	High grade glioma	3.0 T	98	4	2	4
13	74	F	R postero-frontal, parietal	Meningioma	3.0 T	405	3	4	4

a British Medical Research Council Scale (BMRC) for muscle strength.

### Paradigms

Rs-fMRI was performed at the beginning of each scanning session. Subsequently, one or more active tasks were performed depending on lesion location (Table 2). During rs-fMRI, participants were instructed to relax with their eyes open and not to fall asleep. The tb-fMRI paradigms included three motor tasks (hand, foot and mouth movements) and a hand somatosensory stimulation task. All tasks were presented with visual instructions in a blocked-design with 12 active blocks (6 for each side) and 6 rest blocks. All patients practiced the motor tasks before and again just after entering the scanner. During data acquisition task performance was monitored visually.

**Table 2:** Summary of administered tasks, quality of motor performance and available functional maps.

Case	Active task(s)	Affected hemisoma	Performance	tb-fMRI	aROI	fROI	ICA	ECS
1	hand	L	good	√	√	√	√	n.a.
2	hand	L	good	√	√	√	√	n.a.
3	hand	L	failed*	√	-	-	√	hand
4	hand	L	good	√	√	√	√	n.a.
5	hand	L	good	√	√	√	√	n.a.
6	hand	R	good#	√	-	-	-	hand
7	hand/foot/touch	R	good/good/good	√/√/√	√/√	√/√	√	n.a.
8	hand/mouth	L	good/good	√/√	√/√	√/√	√	mouth
9	hand/foot	R	good/weaker	√/√	√/√	√/√	√	n.a.
10	hand/foot/mouth	L	good/good/good	√/−/√	√/√/√	−/√/−	-	hand
11	hand/foot	R	good/weaker	√/√	√/√	√/√	√	hand
12	hand/foot	R	good/good	√/√	√/√	√/√	√	hand and foot
13	hand/foot/mouth	L	failed*/good/good	√/√	√/√	√/√	√	hand and foot

Good denotes satisfactory and symmetrical performance, failed denotes inability to tap using all fingers, weaker denotes ability to tap but more slowly or with smaller movements. Superscript ‘*’ indicates that the patient did not tap but counted by finger extension; ‘#’ indicates that the patient performed the task well with the affected hemisoma but had plegic contralateral hand since birth; ‘√’ and ‘-’ respectively denote maps that were deemed satisfactory and unusable; n.a. indicates not available; task-based fMRI (tb-fMRI); aROI: seed-based analysis using anatomical ROI; fROI: seed-based analysis using functional ROI; ICA: independent-component analysis; electro-cortical stimulation (ECS) responses.

The hand motor task consisted of tapping in sequence all fingers against the thumb, performing repetitive, self-paced, large movements. This task was administered to all patients, since all lesions were close to the hand knob. The foot motor task consisted of alternating flexion and extension and was performed in 6 cases where the lesion extended to the paracentral lobule and to the medial and superior portions of the central sulcus. The mouth motor task consisted of alternating smiling and lip protrusion and was performed in 3 cases where the lesion involved the inferior peri-Sylvian motor area. The hand stimulation task consisted of the rhythmically stroking the ventral hand with a gauze, and was administered in one case with postcentral involvement.

Patients were instructed to maintain a relaxed posture and remain as still as possible, and the head was gently restrained with decompression or foam cushions. Rigid-body image realignment indicated that the peak head displacement was 1.3±0.7 mm during rs-fMRI and 0.7±0.7 mm during tb-fMRI. The corresponding frame-to-frame median displacement was 0.05±0.03 mm during rs-fMRI and 0.11±0.06 mm during tb-fMRI, and did not correlate with the thresholds determined by AMPLE (see below).

### Data Acquisition

Four patients were imaged on a 1.5 T scanner (Magnetom Avanto, Siemens AG, Erlangen, DE) using an 8-channel head coil. For rs-fMRI and tb-fMRI, 200 volumes were acquired through an axial gradient-echo echo-planar sequence having TR = 2,000 ms, TE = 45 ms, α = 90°, 3.5 mm isotropic voxel size, 90×90 matrix size, 26 slices with 10% gap.

Nine patients were imaged on a 3.0 T scanner (Achieva TX, Philips BV, Best, NL) using a 32-channel head coil. For rs-fMRI, 200 volumes were acquired through an axial gradient-echo echo-planar sequence having TR = 2,800 ms, TE = 30 ms, α = 70°, 2.5 mm isotropic voxel size, 90×95 matrix size, 50 slices with 10% gap. For tb-fMRI, the number of volumes was 140, TR = 3,000 ms, TE = 35 ms and 40 slices were acquired. These settings reflected those approved locally for clinical practice.

### Data Analysis

Preprocessing and statistical parametric mapping were performed using SPM8 (Wellcome Trust Center for Neuroimaging, London, UK) running under Matlab 7 (The Mathworks, Natick MA, USA). For both rs-fMRI and tb-fMRI, spatial realignment and slice-timing correction were performed.

For tb-fMRI, statistical analysis was performed in native rather than standard space to remove any risk of error propagation during analysis, since the data were used clinically for presurgical planning. Spatial smoothing was performed using an 8 mm isotropic Gaussian kernel, followed by fixed-effects analysis based on the convolution of task boxcars with the canonical hemodynamic response function, with removal of movement-related nuisance variance. The parametric maps were thereafter spatially normalized using parameters estimated from segmentation of the co-registered anatomical scan; specifically, the standard procedure involved co-registering the mean raw functional volume to the anatomical volume and iteratively segmenting and normalizing the anatomical volume based on SPM8’s built-in template and tissue prior distributions. This procedure can have advantages in respect to direct overlap of functional volumes to an echo-planar imaging atlas because the segmentation provides an accurate normalization of individual anatomy.

For rs-fMRI, the functional volumes were normalized after slice-timing correction, and a brain mask was applied to remove non-parenchymal voxels. Movement-related variance was thereafter removed by multilinear regression, baseline was fitted using a 3^rd^ order polynomial and subtracted, and a 0.1 Hz low-pass filter was applied. Residual global signal fluctuations were regressed out and 8 mm smoothing was performed. The data were then analyzed with three different approaches: i) SBA using a seed region defined exclusively on the basis of contralateral anatomy (anatomical ROI, aROI), ii) SBA using a seed region defined exclusively on the basis of contralateral activation (functional ROI, fROI) and iii) ICA.

Representative orthogonal sections of normalized tb-fMRI and rs-fMRI volumes are provided in Fig. S1. The quality of normalization was assessed by two experienced operators and good matching of the brain outline was attained in all cases.

For the aROI analysis, the pre- and post-central gyri from the automated anatomical labeling atlas [31] were taken as reference, with further manual adjustments to account for anatomical variations. Specifically, the hand sensorimotor area was defined separately for each patient, identifying the knob-like structure on the contralateral hemisphere and choosing the 10 2-mm slices around it in the precentral gyrus for the hand motor area and in the postcentral gyrus for the hand sensory area [24], [32]. The foot motor area was defined as the whole paracentral lobule [33]. The mouth motor area was defined as the ventral portion of the precentral gyrus, ranging from the Sylvian fissure up to the inferior limit of the hand knob [24]. For the fROI analysis, a spherical ROI with 6 mm radius was centered in the contralateral hemisphere around the activation peak obtained with the non-diseased hand or foot [23]. SBA was performed extracting the mean BOLD time-source from each aROI or fROI and entering it as a regressor for fixed-effects analysis.

ICA was performed independently for each patient, using the group ICA of fMRI toolbox (GIFT, MIALab, University of New Mexico, USA) and assuming a fixed number of 20 components [34], [35], [28]. The sensorimotor network was identified upon agreement of two experienced observers, who searched for significant correlation clusters specifically in the pre- and post-central gyri [15]. While ICA is commonly performed without temporal filtering, here it was applied to the same filtered series used for SBA for consistency of comparisons; recent work suggests that, while more robust than SBA to physiological noise, ICA may nevertheless benefit from physiological noise reduction, particularly in terms of unmixing of components [36].

For comparison between the two scanners, the temporal signal-to-noise ratio (tSNR) was calculated from the normalized and realigned resting-state series, calculating mean divided by temporal standard deviation for each voxel and averaging over the whole segmented white matter.

### Threshold Determination

Reflecting common practice in clinical presurgical mapping, the threshold was determined independently by two experienced observers, who adjusted it aiming to get an adequate compromise between activation extent and presence of spurious clusters outside the motor cortex. This operator-dependent step had to be introduced to account for inter-individual differences in activation intensity; it reflects the common procedure used in clinical presurgical fMRI and was used in previous studies in this area [14], [33]. The operators rated each map in isolation, while blinded to all others, viewing in sequence all maps from each method (ICA, aROI, fROI, tb-fMRI) to minimize memory of specific cases. The thresholds chosen by the two observers were averaged, and the inter-rater agreement was 0.83 for tb-fMRI, 0.78 for aROI, 0.89 fROI and 0.71 for ICA.

Since operator-dependence of threshold determination might introduce bias in the comparison between two techniques of different familiarity, yielding potentially different activation patterns, we sought to confirm the results with an automatic threshold determination method, known as activation mapping as percentage of local excitation (AMPLE) [37]. The motor cortex was identified as the pre- and post-central gyri for hand and mouth tasks and as the paracentral lobule for foot tasks. For each patient, task and method (ICA, aROI, fROI, tb-fMRI), the highest t- or z-score in the motor cortex ipsilateral to the lesion was determined, and the threshold was set to 50% of this level.

For the purpose of preoperative planning, only tb-fMRI activation maps generated with the operator-chosen thresholds were considered.

### Determination of Overlap

The topographical overlap between tb- and rs-fMRI maps was calculated as:
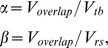
where V_tb_ denotes the number of supra-threshold voxels in tb-fMRI activation map, V_rs_ the number of supra-threshold voxels (see below for thresholding) in rs-fMRI correlation map and V_overlap_ the number of supra-threshold voxels in common between the two. Parameters α and β denote the ratio of common supra-threshold voxels with respect to the number of supra-threshold voxels in tb- and rs-fMRI, respectively. In other words, α can be intended as a measure of sensitivity of rs-fMRI with respect to tb-fMRI: it is 0 if there are no common voxels, and 1 if the supra-threshold voxels in the rs-fMRI map completely enclose the tb-fMRI activation. On the other hand, β is a measure of specificity of rs-fMRI with respect to tb-fMRI: it is 0 if there are no common voxels, and 1 if all supra-threshold voxels in the rs-fMRI map are contained within the tb-fMRI activation.

To improve the anatomical specificity of α and β measurements, for both rs- and tb-fMRI maps we excluded voxels outside the motor cortex, defined as the pre- and post-central gyri for hand and mouth tasks (mask volume 65 ml for right and 73 ml for left hemisphere), and as the paracentral lobule for foot task (mask volume 24 ml for right and 29 ml for left hemisphere). Since several patients had visible mass effect involving the sensorimotor area, the pre- and post-central gyri masks were morphologically dilated by 2 voxels and the paracentral lobule mask was dilated by 6 voxels.

### Centre-of-Mass (CoM) Calculation

For each method, the centre-of-mass (CoM) of the supra-threshold voxels in the motor cortex was calculated as the average of their co-ordinates weighted by the corresponding t-scores. Voxels outside the pre- and post-central gyri or paracentral lobule were discarded, to avoid bias by spurious clusters outside the cortical area of interest. Notably, the CoM was calculated from all supra-threshold voxels, without pre-selecting specific clusters: this removed potential confounds related to “fragmentation” of activity in multiple clusters.

The distances between the CoMs yielded by each rs-fMRI method and tb-fMRI task were then calculated to obtain an objective measure of topographical alignment. Further, the distances between all CoMs and the CoM of the manually-segmented lesion were measured to test for differences between rs-fMRI and tb-fMRI in terms of localization of activity near the lesion borders. For this purpose, the borders of the lesion core were manually traced by a senior neuroradiologist blinded to the functional data and using T1-, T2-weighted and Gadolinium-enhanced images, where available.

### Intraoperative Electro-Cortical Stimulation (ECS)

Electro-cortical stimulation (ECS) data were available for 7 patients who underwent neurophysiological mapping of motor cortex and corticospinal tract during mini-invasive neurosurgery supported by tb-fMRI activation maps [38]. ECS of motor and peri-motor cortex was performed either using a hand-held bipolar probe (60 Hz frequency, 1 ms pulse-width, maximum current 10 mA gradually increased in 1 mA steps) or a 4-contacts strip electrode (5 pulse-trains at 200 Hz rate, 1 ms pulse width, maximum current 25 mA). Motor responses were collected by surface electromyography (EMG), recording the wrist extensor, deltoid, abductor pollicis brevis and abductor digiti minimi for patients stimulated in the hand knob, the rectus femori, tibialis anterior and extensor digitorum brevi for patients stimulated around the foot area, and the orbicularis oris for patients stimulated in the mouth area.

Mapping of tissue not activated by tb-fMRI was also performed, but in a few sites for patient safety. Therefore agreement between ECS and fMRI was assessed on stimulation points which elicited motor responses on EMG. This allowed us to have additional information about the precision of tb-fMRI and rs-fMRI, without, however, calculating sensitivity and specificity values [4].

The position of each cortical stimulation site was determined with the aid of the neuronavigation system as well as on the basis of the anatomic location of sulci and gyri [7], [23]. Stimulation sites that were on the dura mater were Cartesian projected onto the cortical surface and the shortest distance from the fMRI activation was calculated for each method. fMRI and ECS were considered to match when the fMRI activation was within the immediate vicinity (<7–10 mm) of intraoperative ECS sites, in line with previous studies [4], [10], [12], [13], [39].

## Results

### Map Availability, Threshold Choice and Signal-to-noise Ratio

Active task performance, availability of tb- and rs-fMRI maps and ECS data are summarized in Table 2. All patients performed at least one task satisfactorily. One patient had peripheral hemiplegia affecting the hemisoma contralateral to the side affected by the primary lesion (left hemiplegia and left hemisphere lesion) and did not yield usable rs-fMRI data (case 6); further, rs-fMRI maps from some aROI, fROI and ICA analyses were deemed un-informative and rejected. The remaining tb- and rs-fMRI maps were all spatially consistent with the known topography of the sensorimotor network [15], [16], [20], [21], [25], [40], [41].

Thresholds manually determined upon agreement of the two expert operators and thresholds automatically determined using AMPLE are given separately for patients scanned at 1.5 T and 3.0 T in Table 3. While the difference between 1.5 T and 3.0 T on the thresholds for tb-fMRI (hand task only, as this was performed by all patients) and ICA was relatively constrained, the thresholds for fROI and aROI were considerably higher at 3.0 T than 1.5 T. As regards the tSNR, it was lower at 3.0 T (160±11) than 1.5 T (194±2) due to difference in voxel size (16 mm^3^ at 3.0 T and 43 mm^3^ at 1.5 T). Statistical comparisons between the two field strengths were not performed due to limited numerosity.

**Table 3:** Chosen thresholds for each method and scanner.

Chosen threshold (t-score)	tb-fMRI	aROI	fROI	ICA
Operator-dependent, 1.5 T	6.4±2.3	5.0±1.0	3.3±1.8	2.3±0.9
AMPLE, 1.5 T	6.8±2.6	3.9±0.8	3.7±0.5	3.2±1.3
Operator-dependent, 3.0 T	6.4±2.6	7.4±1.8	7.4±2.6	1.8±0.4
AMPLE, 30 T	6.8±2.6	8.1±3.5	8.0±4.2	2.9±1.2

Values are given as mean ± standard deviation. Task-based fMRI (tb-fMRI); aROI: seed-based analysis using anatomical ROI; fROI: seed-based analysis using functional ROI; ICA: independent-component analysis.

As regards the difference between operator-chosen and AMPLE thresholds, results were very similar (see below) and linear regression revealed strong correlation for tb-fMRI (r = 0.87, p<0.001), aROI (r = 0.83, p<0.001) and fROI (r = 0.71, p = 0.001), but not ICA (p = 0.2).

### Topographical Overlap

Based on thresholds chosen manually by the operators (Table 4), the sensitivity of rs-fMRI with respect to tb-fMRI was comparable between aROI, fROI and ICA, with α∼0.43 and no significant differences (ANOVA p = 1); by contrast, there were significant differences in specificity (ANOVA F(2,34) = 9.4, p<0.001), as it was significantly higher for fROI than aROI and ICA (β∼0.51 vs. 0.30 p_Bonf_<0.005 and 0.34, p_Bonf_<0.05).

**Table 4:** Activation volumes (calculated over the corresponding anatomical masks), overlap sensitivity (α) and specificity (β) of resting-state fMRI (rs-fMRI) with respect to task-based fMRI (tb-fMRI), obtained with operator-dependent thresholds.

	Activation volume/ml				aROI		fROI		ICA	
Case	tb-fMRI	aROI	fROI	ICA	α	β	α	β	α	β
**Hand motor task**										
1	8.3	7.0	2.6	11.2	0.54	0.64	0.26	0.86	0.95	0.71
2	8.1	8.4	6.6	10.2	0.26	0.26	0.34	0.42	0.27	0.22
3	6.5	-	-	14.2	-	-	-	-	0.06	0.03
4	9.7	1.1	2.9	16.6	0.03	0.29	0.27	0.90	0.68	0.40
5	5.7	21.6	12.1	11.2	0.87	0.23	0.78	0.37	0.69	0.35
6	7.9	-	-	-	-	-	-	-	-	-
7	7.4	5.2	3.2	10.8	0.10	0.15	0.35	0.83	0.62	0.43
8	7.7	4.6	2.9	10.8	0.12	0.19	0.08	0.21	0.23	0.17
9	5.3	17.6	15.0	15.7	0.81	0.24	0.75	0.27	0.81	0.27
10	4.3	3.4	-	-	0.22	0.28	-	-	-	-
11	6.7	17.1	16.3	7.1	0.72	0.28	0.76	0.31	0.38	0.36
12	5.8	25.9	6.8	10.4	0.93	0.21	0.68	0.59	0.11	0.06
13	7.4	6.6	1.5	15.3	0.14	0.16	0.06	0.32	0.00	0.00
*Hand mean*	*7.0*	*10.8*	*7.0*	*12.1*	*0.43*	*0.27*	*0.44*	*0.51*	*0.44*	*0.27*
*SD*	*1.5*	*8.3*	*5.5*	*2.9*	*0.35*	*0.13*	*0.28*	*0.27*	*0.33*	*0.21*
**Foot motor task**										
7	3.1	7.4	3.5	5.5	0.73	0.30	0.60	0.54	0.63	0.35
9	3.7	4.4	4.2	3.1	0.54	0.46	0.44	0.39	0.39	0.46
10	-	8.1	2.7	-	- -	- -	- -	- -	-	-
11	2.7	4.5	5.3	0.8	0.52	0.31	0.56	0.28	0.04	0.14
12	2.4	8.9	6.0	0.3	0.90	0.24	0.78	0.31	0.09	0.68
13	10.3	9.0	3.5	2.4	0.31	0.36	0.18	0.54	0.11	0.48
*Foot mean*	*4.4*	*7.0*	*4.2*	*2.4*	*0.60*	*0.33*	*0.51*	*0.41*	*0.25*	*0.42*
*SD*	*3.3*	*2.1*	*1.2*	*2.1*	*0.22*	*0.08*	*0.22*	*0.12*	*0.25*	*0.20*
**Mouth motor task**										
8	8.8	10.5	4.6	10.8	0.41	0.34	0.41	0.77	0.58	0.47
10	6.0	8.4	-	-	0.08	0.05	-	-	-	-
13	14.1	8.0	11.1	15.3	0.33	0.58	0.40	0.52	0.49	0.45
**Hand stimulation task**										
7	6.6	1.4	3.5	10.8	0.10	0.48	0.45	0.85	0.65	0.39
**Tot mean**	**6.8**	**9.0**	**6.0**	**9.6**	**0.43**	**0.30**	**0.45**	**0.51**	**0.41**	**0.34**
**SD**	**2.7**	**6.4**	**4.4**	**5.1**	**0.31**	**0.14**	**0.24**	**0.23**	**0.29**	**0.20**

Sign ‘-’ denotes unavailable maps; ‘- - ’ denotes unavailable α and β calculation due to lack of tb-fMRI map (see Table 2); aROI: seed-based analysis using anatomical ROI; fROI: seed-based analysis using functional ROI; ICA: independent-component analysis.

Considering the hand motor task only (13 cases), the sensitivity was comparable between aROI, fROI and ICA (α∼43) with no significant differences (ANOVA p = 1); by contrast the specificity was significantly higher for fROI than aROI and ICA (β∼0.51 vs. 0.27, p_Bonf_<0.05 and 0.27, p_Bonf_<0.05).

Considering the foot task only (5 of the 6 cases available), the higher sensitivity was obtained with aROI with respect to fROI and ICA (α∼0.60 vs. 0.51 and 0.25, respectively), without significant differences; the higher specificity was obtained with ICA, with respect to aROI and fROI (β∼0.42 vs. 0.33 and 0.41, respectively), without significant differences. The latter result should be interpreted with caution: as indicated by the activation volumes reported in Table 4, ICA tended to provide larger activation volumes in comparison to tb-fMRI for hand and mouth tasks, but only a very small number of voxels on ICA maps intersected with the foot area.

Based on the AMPLE criterion (Table 5), overlap sensitivity and specificity were similar to those obtained with the operator-dependent method. The sensitivity was α∼0.40 and was significantly higher for fROI than ICA (α∼0.46 vs 0.31, p_Bonf_<0.05), whereas specificity was β∼0.46 and was significantly higher for fROI than aROI (β∼0.56 vs 0.38, p_Bonf_<0.005).

**Table 5:** Activation volumes (calculated over the corresponding anatomical masks), overlap sensitivity (α) and specificity (β) of resting-state fMRI (rs-fMRI) with respect to task-based fMRI (tb-fMRI), obtained with AMPLE thresholds.

	Activation volume/ml				aROI		fROI		ICA	
Case	tb-fMRI	aROI	fROI	ICA	α	β	α	β	α	β
**Hand motor task**										
1	6.8	12.6	12.4	4.3	0.85	0.45	0.84	0.46	0.58	0.91
2	8.9	14.6	8.6	8.7	0.47	0.29	0.40	0.41	0.24	0.25
3	10.0	-	-	4.9	-	-	-	-	0.10	0.20
4	8.6	10.8	6.2	8.1	0.44	0.35	0.54	0.75	0.43	0.46
5	5.9	10.2	4.9	5.0	0.64	0.37	0.50	0.60	0.41	0.49
6	6.2	-	-	-	-	-	-	-	-	-
7	8.8	15.2	6.9	11.4	0.37	0.21	0.51	0.65	0.61	0.47
8	6.4	9.0	8.2	5.5	0.25	0.18	0.25	0.19	0.07	0.09
9	4.5	13.8	14.7	3.7	0.75	0.25	0.78	0.24	0.33	0.40
10	4.0	7.5	-	-	0.50	0.27	-	-	-	-
11	3.2	9.6	9.7	9.5	0.59	0.19	0.73	0.24	0.75	0.25
12	4.9	15.6	4.9	3.3	0.87	0.27	0.60	0.59	0.00	0.00
13	9.4	6.9	7.2	3.7	0.17	0.22	0.32	0.41	0.00	0.00
*Hand mean*	*6.7*	*11.4*	*8.4*	*6.2*	*0.53*	*0.28*	*0.54*	*0.45*	*0.32*	*0.32*
*SD*	*2.2*	*3.1*	*3.2*	*2.7*	*0.23*	*0.08*	*0.19*	*0.19*	*0.26*	*0.27*
**Foot motor task**										
7	3.5	5.9	2.8	5.0	0.64	0.38	0.47	0.59	0.55	0.39
9	8.3	3.2	2.3	3.3	0.26	0.68	0.20	0.70	0.26	0.65
10	-	3.2	4.4	-	- -	- -	- -	- -	-	-
11	6.0	2.7	1.2	1.9	0.26	0.57	0.14	0.70	0.18	0.55
12	7.3	3.3	4.9	0.8	0.30	0.67	0.43	0.64	0.08	0.72
13	18.3	9.0	8.0	1.2	0.27	0.55	0.27	0.61	0.06	0.89
*Foot mean*	*8.7*	*4.5*	*3.9*	*2.4*	*0.35*	*0.57*	*0.30*	*0.65*	*0.22*	*0.64*
*SD*	*5.7*	*2.5*	*2.4*	*1.7*	*0.17*	*0.12*	*0.14*	*0.05*	*0.20*	*0.19*
**Mouth motor task**										
8	4.6	6.0	2.3	5.5	0.46	0.35	0.44	0.86	0.52	0.43
10	8.6	5.9	-	-	0.06	0.09	-	-	-	-
13	27.6	10.4	4.8	3.7	0.30	0.81	0.13	0.73	0.09	0.67
**Hand stimulation task**										
7	6.5	2.3	6.8	11.4	0.15	0.41	0.70	0.67	0.66	0.38
**Tot mean**	**8.1**	**8.5**	**6.5**	**5.3**	**0.43**	**0.38**	**0.46**	**0.56**	**0.31**	**0.43**
**SD**	**5.4**	**4.3**	**3.5**	**3.1**	**0.23**	**0.19**	**0.22**	**0.19**	**0.25**	**0.27**

Sign ‘-’ denotes unavailable maps; ‘- - ’ denotes unavailable α and β calculation due to lack of tb-fMRI map (see Table 2); aROI: seed-based analysis using anatomical ROI, fROI: seed-based analysis using functional ROI, ICA: independent-component analysis.

Considering the hand motor task, the sensitivity was higher for fROI than ICA (α∼0.54 vs 0.32, p_Bonf_<0.05), and there were no significant differences for specificity. Compared to the operator-dependent threshold, specificity for the hand motor task increased for aROI and fROI, but decreased for ICA.

Considering the foot motor task, AMPLE selected very stringent thresholds for rs-fMRI and only voxels in the supplementary motor area survived; thus, activation in the paracentral lobule was absent or minimal (see Representative cases below with examples of maps generated with operator-chosen and AMPLE thresholds).

### Centre-of-Mass (CoM) Distances

Based on thresholds chosen manually by the operators (Table 6), the CoM distance between rs-fMRI (aROI, fROI and ICA) and tb-fMRI was measured. There was a non-significant trend towards statistical difference in the distance to tb-fMRI (ANOVA F(2,34) = 3.1, p = 0.08).

**Table 6:** Centre-of-mass (CoM) distance measurements obtained with operator-dependent thresholds.

	Distance to tb-fMRI CoM/mm			Distance to lesion CoM/mm			
Case	aROI	fROI	ICA	tb-fMRI	aROI	fROI	ICA
**Hand motor task**							
1	2.0	2.8	0.0	36.7	35.8	37.6	36.7
2	14.6	9.2	13.4	43.2	35.0	36.8	34.1
3	-	-	37.7	30.6	-	-	47.7
4	21.3	4.5	7.5	39.0	21.9	37.7	32.7
5	4.0	2.0	2.8	35.4	31.6	36.1	33.6
6	-	-	-	29.7	-	-	-
7	10.0	6.0	6.6	28.7	26.2	23.7	25.1
8	2.8	4.5	21.0	51.6	50.2	50.4	34.4
9	15.7	16.4	14.0	34.2	41.7	43.4	41.3
10	16.7	-	-	23.2	30.6	-	-
11	16.4	15.7	16.6	34.1	45.4	44.2	45.7
12	9.2	4.9	36.1	22.2	26.7	20.5	52.5
13	8.5	6.3	33.3	19.8	14.7	14.1	50.9
*Hand mean*	*11.0*	*7.2*	*17.2*	*33.0*	*32.7*	*34.5*	*39.5*
*SD*	*6.4*	*5.1*	*13.4*	*8.8*	*10.4*	*11.4*	*8.7*
**Foot motor task**							
7	3.5	4.9	4.9	36.1	32.8	32.9	32.2
9	10.8	13.1	14.1	41.1	34.2	33.8	33.3
10	- -	- -	-	-	32.2	34.1	-
11	10.2	8.9	15.7	27.0	22.4	21.0	21.4
12	10.8	10.8	15.4	14.7	9.2	9.2	14.6
13	8.9	8.2	12.8	27.5	20.1	22.1	38.3
*Foot mean*	*8.8*	*9.2*	*12.6*	*29.3*	*25.2*	*25.5*	*28.0*
*SD*	*3.1*	*3.1*	*4.5*	*10.1*	*9.8*	*9.9*	*9.7*
**Mouth motor task**							
8	16.2	6.0	2.0	34.2	47.2	31.9	34.4
10	26.3	-	-	44.7	38.8	-	-
13	6.6	7.5	7.5	49.1	45.1	50.9	50.9
**Hand stimulation task**							
7	11.5	2.8	6.3	26.9	33.3	26.3	25.1
**Tot mean**	**11.3**	**7.5**	**14.1**	**33.2**	**32.2**	**31.9**	**36.0**
**SD**	**6.3**	**4.2**	**11.1**	**9.4**	**10.7**	**11.5**	**10.4**

The 3 columns on the left denote the distance between the CoM of task-based fMRI (tb-fMRI) and that measured in the resting-state fMRI (rs-fMRI) maps. The 4 columns on the right denote the distance between the CoM from tb-fMRI and rs-fMRI maps and the lesion border. All values are expressed in mm. Sign ‘-’ denotes unavailable maps; ‘- -’ denotes unavailable distance calculation for lack of tb-fMRI map (see Table 2); aROI: seed-based analysis using anatomical ROI; fROI: seed-based analysis using functional ROI; ICA: independent-component analysis.

The CoM distance between functional maps and lesion was measured. The distance to lesion was comparable between tb-fMRI, aROI, fROI and ICA, ∼33 mm, without significant differences (ANOVA p = 1).

Based on the AMPLE criterion (Table 7), the CoM distances were very similar to those obtained with the operator-dependent threshold. There were no significant differences in the distance to tb-fMRI (p = .1), nor to the lesion (p = .7).

**Table 7:** Centre-of-mass (CoM) distance measurements with AMPLE thresholds.

	Distance to tb-fMRI CoM/mm			Distance to lesion CoM/mm			
Case	aROI	fROI	ICA	tb-fMRI	aROI	fROI	ICA
**Hand motor task**							
1	2.8	2.8	2.8	36.7	35.4	35.4	36.3
2	12.3	9.2	14.7	43.2	34.0	36.8	34.2
3	-	-	29.0	33.1	-	-	49.2
4	14.7	0.0	4.9	37.2	34.3	37.2	32.9
5	4.5	4.9	2.8	38.5	40.5	41.3	40.0
6	-	-	-	29.7	-	-	-
7	8.9	7.5	6.6	28.7	30.3	23.2	25.1
8	4.5	4.9	22.2	51.6	50.6	48.6	34.7
9	19.3	18.2	13.4	33.3	43.3	44.4	41.8
10	14.7	-	-	24.5	29.5	-	-
11	18.3	16.6	16.2	79.0	86.9	86.9	86.4
12	4.5	6.6	39.4	22.2	23.2	20.8	55.8
13	8.5	2.8	32.7	19.8	14.7	19.0	49.8
*Hand mean*	*10.3*	*7.4*	*16.8*	*36.7*	*38.4*	*39.3*	*44.2*
*SD*	*5.9*	*5.9*	*12.6*	*15.4*	*18.8*	*19.5*	*16.6*
**Foot motor task**							
7	2.0	2.8	6.0	36.1	35.5	35.4	34.6
9	10.4	10.8	10.4	37.7	34.6	33.5	34.6
10	- -	- -	-	-	32.2	33.3	-
11	9.2	11.7	12.3	49.3	41.8	38.9	38.7
12	10.2	5.7	11.5	8.7	12.2	10.4	14.7
13	11.0	8.2	13.4	27.0	18.3	20.9	39.7
*Foot mean*	*9.5*	*7.9*	*17.1*	*32.4*	*32.2*	*32.5*	*41.5*
*SD*	*4.5*	*4.2*	*10.5*	*19.3*	*19.8*	*20.4*	*20.4*
**Mouth motor task**							
8	12.8	10.2	6.0	34.2	45.1	30.6	34.7
10	24.8	-	-	43.9	38.8	-	-
13	7.2	10.8	10.8	47.0	45.1	49.8	49.8
**Hand stimulation task**							
7	14.1	4.9	6.6	25.1	35.3	25.6	25.1
**Tot mean**	**10.7**	**7.7**	**13.8**	**35.8**	**36.3**	**35.4**	**39.9**
**SD**	**5.9**	**4.8**	**10.3**	**14.1**	**15.3**	**16.3**	**14.9**

The 3 columns on the left denote the distance between the CoM of task-based fMRI (tb-fMRI) and that measured in the resting-state fMRI (rs-fMRI) maps. The 4 columns on the right denote the distance between the CoM from tb-fMRI and rs-fMRI maps and the lesion border. All values are expressed in mm. Sign ‘-’ denotes unavailable maps; ‘- -’ denotes unavailable distance calculation for lack of tb-fMRI map (see Table 2); aROI: seed-based analysis using anatomical ROI; fROI: seed-based analysis using functional ROI; ICA: independent-component analysis.

### Representative Cases

**Figure 1:**
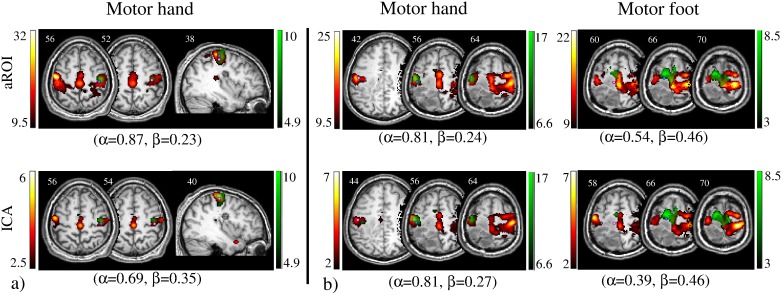
Concordance between task-based (tb-fMRI, in green) and resting-state (rs-fMRI, in red) fMRI maps computed with aROI (top row) and ICA (bottom row). Overlap sensitivity (α) and specificity (β) of rs-fMRI with respect to tb-fMRI, obtained with operator-dependent thresholds, are reported. Light blue circle represents the Centre of Mass (CoM) of tb-fMRI, and pink circle represents the CoM of rs-fMRI. Images are shown in neurological convention (left is left) and MNI coordinates are reported on top of each slice. a) For Case 5 concordance was optimal in terms of overlap values and CoM distance. b) For Case 9 the aROI and ICA maps were extremely similar to each other, in particular for the hand area. However, the aROI was more useful than ICA to localize the foot area as it included the paracentral lobule.

**Figure 2:**
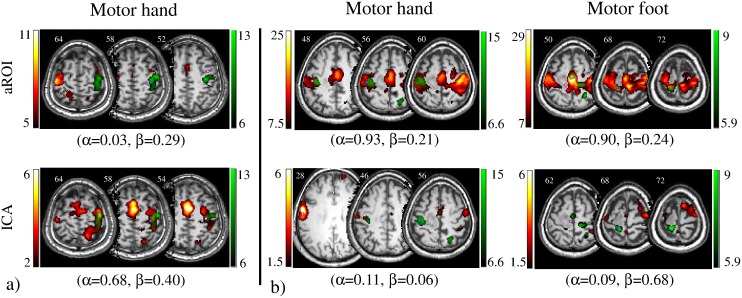
Concordance between task-based (tb-fMRI, in green) and resting-state (rs-fMRI, in red) fMRI maps computed with aROI (top row) and ICA (bottom row). Overlap sensitivity (α) and specificity (β) of rs-fMRI with respect to tb-fMRI, obtained with operator-dependent thresholds, are reported. Light blue circle represents the Centre of Mass (CoM) of tb-fMRI, and pink circle represents the CoM of rs-fMRI. Images are shown in neurological convention (left is left) and MNI coordinates are reported on top of each slice. a) For Case 4 concordance between rs-fMRI and tb-fMRI was better with ICA, while (b) for Case 12 was better with aROI.

**Figure 3:**
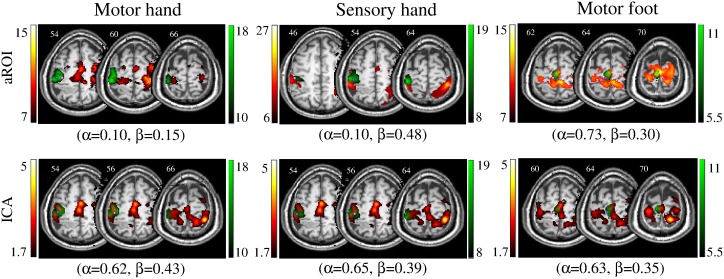
Concordance between task-based (tb-fMRI, in green) and resting-state (rs-fMRI, in red) fMRI maps computed with aROI (top row) and ICA (bottom row). Overlap sensitivity (α) and specificity (β) of rs-fMRI with respect to tb-fMRI, obtained with operator-dependent thresholds, are reported. Light blue circle represents the Centre of Mass (CoM) of tb-fMRI, and pink circle represents the CoM of rs-fMRI. Images are shown in neurological convention (left is left) and MNI coordinates are reported on top of each slice. For Case 7, ICA showed better overlap values for the motor and the sensory hand area, while aROI showed better overlap values for the foot area. The CoM distance task-ICA was the shortest (<7 mm) for the hand and CoM distance task-aROI was the shortest (<4 mm) for the foot.

Example tb- and rs-fMRI maps from representative cases are shown in Fig. 1–3. Mirroring the numerical results reported in Tables 4 and 6 (with operator-dependent thresholds), concordance between rs- and tb-fMRI varied among cases. In case 5 (Fig. 1a) concordance was strongest: overlap with tb-fMRI was high for aROI as well as for ICA and the CoM distance with respect to tb-fMRI was <5 mm for both aROI and ICA. In case 9 (Fig. 1b) the aROI and ICA maps were very similar to each other, in particular for the hand area. However, the aROI proved more useful than ICA to localize the foot area as it included significant voxels in the paracentral lobule. In case 4 (Fig. 2a) concordance between rs fMRI and tb-fMRI was better for ICA than SBA, while in case 12 (Fig. 2b) it was better for aROI. In case 7 (Fig. 3) ICA showed better overlap for the motor and the sensory hand area, while aROI showed better overlap for the foot area. The CoM distance with respect to tb-fMRI was shortest with ICA (<7 mm) for the hand, and with aROI (<4 mm) for the foot.

**Figure 4:**
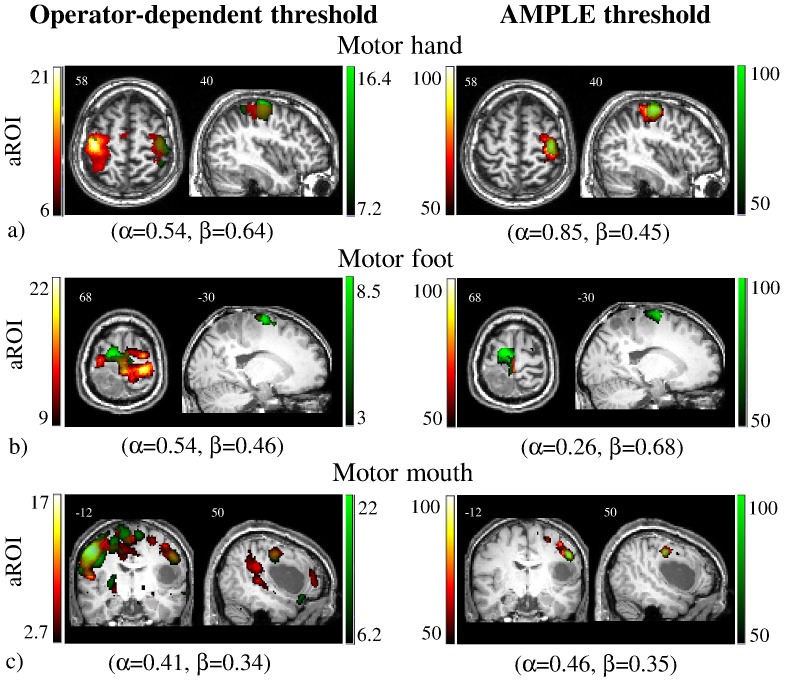
Example of direct comparison between the thresholds chosen by two operators and the thresholds determined by AMPLE. Concordance between task-based (tb-fMRI, in green) and resting-state (rs-fMRI, in red) fMRI maps computed with aROI. Overlap sensitivity (α) and specificity (β) of rs-fMRI with respect to tb-fMRI are reported. a) For case 1 mapping of the hand area with AMPLE was satisfactory and aROI sensitivity increased with respect to the operator-dependent threshold. b) For case 9 mapping of the foot area with AMPLE was good with tb-fMRI, while with aROI the paracentral lobule was not activated and aROI sensitivity decreased with respect to the operator-dependent threshold. c) In case 8 mapping of the mouth area with AMPLE was successful with both tb-fMRI and rs-fMRI and results were similar to those obtained with the operator-dependent threshold. Images are shown in neurological convention (left is left) and MNI coordinates are reported on top of each slice.

Examples of tb- and rs-fMRI maps generated with operator-chosen and AMPLE thresholds are reported in Fig. 4. Compared to the operator-dependent threshold, the AMPLE criterion produced maps which localized the hand and mouth areas successfully, both with tb-fMRI and rs-fMRI data; on the contrary, AMPLE localized the foot area satisfactorily with tb-fMRI, but not with rs-fMRI. In case 1 (Fig. 4a) mapping of the hand area with AMPLE was good and aROI sensitivity increased with respect to the operator-dependent threshold (α = 0.85 vs. α = 0.54). In case 9 (Fig. 4b) mapping of the foot area with AMPLE was adequate with tb-fMRI, while with aROI the paracentral lobule was not activated and aROI sensitivity decreased with respect to the operator-dependent threshold (α = 0.54 vs. α = 0.26). In case 8 (Fig. 4c) mapping of the mouth area with AMPLE was successful with both tb-fMRI and rs-fMRI and results were similar to those obtained with the operator-dependent threshold.

### ECS Results

**Figure 5:**
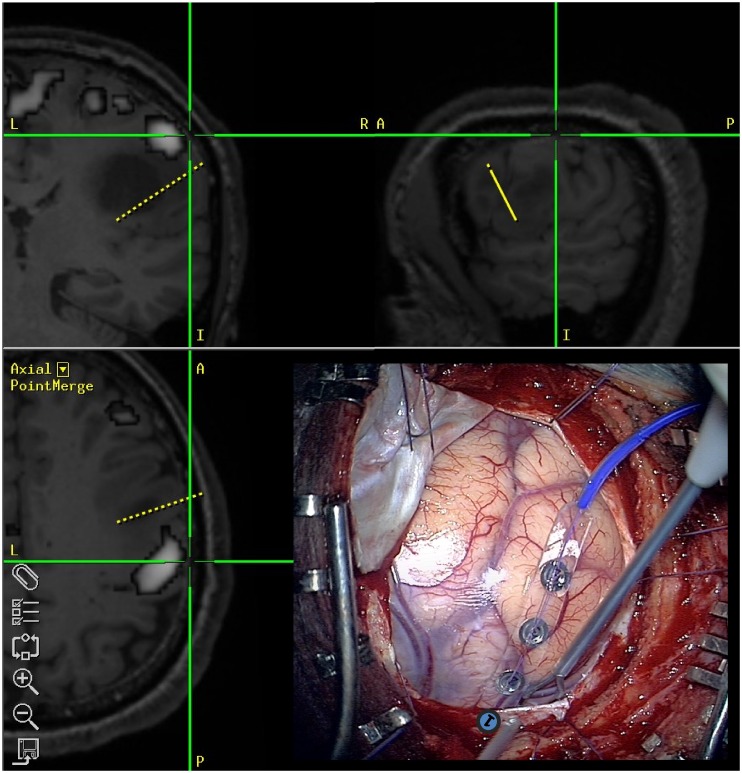
A representative case (Case 8) of intraoperative electro-cortical stimulation (ECS) mapping, performed through the use of a bipolar probe and a 4-contacts strip electrode (bottom right), with the support of neuronavigation system. Task-based fMRI map overlaid on T1-weighted MR images was loaded on the neuronavigation device (upper panel and bottom left). In this patient, ECS evoked mouth motion in the cortical site 1 (under the dura), as illustrated in intraoperative optic microscopic view (bottom right). With the aid of the neuronavigation device the position of each ECS site was determined on the presurgical fMRI dataset.

ECS data were available for 7 patients with a total of 13 cortical stimulation sites (Table 8, Fig. 5). As regards the thresholds chosen manually by the operators, all stimulation points which elicited motor responses were included in or adjacent to tb-fMRI activations (mean distance 1.8 mm±2.4 mm). For rs-fMRI, the shortest distance from the ECS points was obtained with aROI (mean 2.7±2.7 mm), followed by fROI (mean 4.2±6.0 mm) and by ICA (8.3±7.3 mm). Differences between the 4 techniques were not statistically significant (ANOVA p = 0.33). The proportion of stimulation sites with distance to the nearest activated voxel <10 mm was 100% for tb-fMRI and aROI, 75% for fROI and 60% for ICA.

**Table 8:** Agreement between electro-cortical stimulation (ECS) sites and fMRI activation border obtained with operator-dependent thresholds for 13 points in 7 patients.

Case	Point	ECS coord/mm	Motor response	Distance to fMRI activation border/mm			
				tb-fMRI	aROI	fROI	ICA
3	1	22–24 74	hand	0	-	-	17.9
	2	20–18 78	hand	0	-	-	20.4
6	1	−36–20 68	hand	0	-	-	-
8	1	64–4 28	mouth	2	2	2	0
10	1	40–26 66	hand	4.9	7.2	-	-
	2	40–14 64	hand	6	4	-	-
11	1	−28–16 72	hand	2	0	0	2.8
12	1	−40–22 68	hand	0	0	0	2
	2	−2–24 68	foot	6.3	0	0	8.2
13	1	42–10 64	hand	2	5.7	14.1	2
	2	40–10 64	hand	0	4.5	12.8	4
	3	12–22 78	foot	0	4	4.9	11.8
	4	16–18 76	foot	0	0	0	14.1
**N**				**13**	**10**	**8**	**10**
**Mean**				**1.8**	**2.7**	**4.2**	**8.3**
**SD**				**2.4**	**2.7**	**6.0**	**7.3**
**ECS sites<10 mm**				**100%**	**100%**	**75%**	**60%**
**ECS sites<7 mm**				**100%**	**90%**	**75%**	**50%**

Sign ‘-’ denotes absence of a usable fMRI map; value ‘0’ indicates that the stimulation point was inside the activation; MNI coordinates of the ECS site (ECS coord) are reported. Task-based fMRI (tb-fMRI); aROI: seed-based analysis using anatomical ROI; fROI: seed-based analysis using functional ROI; ICA: independent-component analysis.

Based on the AMPLE criterion (Table 9), results were similar to those obtained with the operator-dependent threshold. All stimulation points were included in or adjacent to tb-fMRI activation clusters (mean distance 2.8 mm±1.9 mm). For rs fMRI, the shortest distance from the ECS points was obtained with aROI (mean 3.5±1.7 mm), followed by fROI (mean 4.1±2.8 mm) and by ICA (20.4±14.3 mm). There was a significant main effect among distances (ANOVA F(3,21) = 9.6, p<0.005), with a trend for ICA distance being longer than tb-fMRI (p = .09) and aROI distances (p = .09). The proportion of stimulation sites with distance to the nearest activated voxel <10 mm was 100% for tb-fMRI, aROI and fROI, and 30% for ICA.

**Table 9:** Agreement between electro-cortical stimulation (ECS) sites and fMRI activation border obtained with thresholds determined by AMPLE for 13 points in 7 patients.

Case	Point	ECS coord/mm	Motor response	Distance to fMRI activation border/mm			
				tb-fMRI	aROI	fROI	ICA
3	1	22–24 74	hand	4.5	-	-	38
	2	20–18 78	hand	2.8	-	-	41.4
6	1	−36–20 68	hand	2	-	-	-
8	1	64–4 28	mouth	3.4	2	2	0
10	1	40–26 66	hand	4.9	4.5	-	-
	2	40–14 64	hand	6.6	4	-	-
11	1	−28–16 72	hand	4.8	4.5	4.9	2.8
12	1	−40–22 68	hand	2	2	2	30.8
	2	−2–24 68	foot	0	0	0	7.2
13	1	42–10 64	hand	2	5.7	8.2	24.5
	2	40–10 64	hand	0	4.5	7.4	26
	3	12–22 78	foot	2	4	4	16.5
	4	16–18 76	foot	2	4	4	16.5
**N**				**13**	**10**	**8**	**10**
**Mean**				**2.8**	**3.5**	**4.1**	**20.4**
**SD**				**1.9**	**1.7**	**2.8**	**14.3**
**ECS sites<10 mm**				**100%**	**100%**	**100%**	**30%**
**ECS sites<7 mm**				**100%**	**100%**	**75%**	**20%**

Sign ‘-’ denotes absence of a usable fMRI map; value ‘0’ indicates that the stimulation point was inside the activation; MNI coordinates of the ECS site (ECS coord) are reported. Task-based fMRI (tb-fMRI); aROI: seed-based analysis using anatomical ROI; fROI: seed-based analysis using functional ROI; ICA: independent-component analysis.

## Discussion

We investigated the potential utility of rs-fMRI as a presurgical mapping tool in a group of 13 patients with lesions close to the sensorimotor cortex, and assessed quantitatively the degree of correspondence between multiple rs-fMRI analysis techniques and tb-fMRI, with reference to sensitivity and specificity of rs-fMRI with respect to tb-fMRI, and CoM location. Correspondence with ECS mapping data was also investigated.

Quantitative measurements and visual observation (Fig. 1–3) confirmed that rs-fMRI can localize the sensorimotor cortex successfully. In particular, considering all three analyses techniques together (23 aROI, 23 fROI, 13 ICA), rs-fMRI provided informative maps in 86% of cases (Table 2), even when tb-fMRI failed (foot motor task in case 10). This is in line with previous studies showing a good identification of the sensorimotor area with rs-fMRI [20], [23]–[25]. While ICA maps extracted the whole sensorimotor component, SBA maps obtained with aROI and fROI approaches showed some anatomical specificity to the motor subregion of interest, as they could distinguish hand, foot and mouth motor areas and hand sensory area (Fig. 1–3). This is consistent with a previous report by Liu et al. [24], showing selectivity for hand and tongue regions with rs-fMRI.

In terms of distance between significant voxels and the lesion CoM, rs-fMRI and tb-fMRI yielded comparable results (Table 8). Importantly, the CoM distance is not influenced by the size of the activation pattern. ECS mapping data showed that significant voxels on the rs-fMRI maps were relatively close to the stimulations sites that evoked motor responses; the distance was on average shortest for aROI maps (mean 2.7 mm). Further, all stimulated sites evoking a response were within 10 mm of significant voxels for both tb-fMRI and aROI. Taken together, these indices confirmed good accuracy in localizing relevant motor regions.

However, our results do not suggest that rs-fMRI can be considered as an outright alternative to tb-fMRI for presurgical planning. We observed substantial variability in the correspondence between tb-fMRI and rs-fMRI maps across cases. Overlap sensitivity and specificity of rs-fMRI with respect to tb-fMRI were not high, on average α∼0.43 and β∼0.30–0.51, respectively. The main difference is that localization performed by the two approaches can include different portions of the sensorimotor area and that the activation pattern is typically larger for rs-fMRI than tb-fMRI, in particular with ICA (Table 4). This might lead to a greater portion of false positives [20] and reduced number of false negatives [22], in either case ultimately affecting the extent of resection, if fMRI is not used in conjunction with ECS.

The CoM distance between rs-fMRI and tb-fMRI maps was ∼11 mm. Previous studies which measured the CoM variation between repeated sessions of a hand motor task reported a CoM distance of 3–6.2 mm [8], [42], [43]. This confirms that localization performed by rs-fMRI and tb- fMRI gives substantially different results, partially in contrast with previous reports concluding that rs-fMRI yields quite similar or even better results than tb-fMRI [23], [24]. It should be noted that in previous studies the co-localization of activations in sensorimotor areas was assessed qualitatively and on a smaller number of cases. Kokkonen et al. [25] showed with a quantitative analysis that ICA on rs-fMRI data can localize the sensorimotor area in brain-tumor patients in the same way as in healthy subjects. However, the analysis was performed between groups, not between techniques (ICArest, ICAtask, tb-fMRI) which were compared only qualitatively.

The design of the present study hinged around tb-fMRI as a reference technique, with respect to which the sensitivity and specificity of rs-fMRI were measured. This design aspect requires careful consideration, given that tb-fMRI is by no means a gold-standard reference of activity localization. In particular, tb-fMRI is only able to detect correlations between brain activity and task performance; unlike ECS, it cannot conclusively determine whether a given area is functionally involved in performing a given task [44]. Furthermore, irrespective of the fact that resting-state and task-evoked neural activity can represent different processes and be uncoupled from one another, there are common vascular confounds which may similarly affect the signals measured using rs-fMRI and tb-fMRI, introducing potential circularity in comparisons between the two techniques [45], [46]. For these reasons, the comparison with tb-fMRI should be viewed not as a formal validation of rs-fMRI, but rather as a pragmatic comparison between a relatively novel localization technique and one that has been around for approximately two decades and with which the majority of neurosurgeons and neuroradiologists are presently familiar.

Functional connectivity can artefactually decrease in presence of neoplastic lesions and associated edema, due to vascular and metabolic changes that lead to neurovascular uncoupling. It is well-known that tumor vasculature responds less vigorously to physiological stimuli and spontaneous neural activity fluctuations than vessels in normal cerebral cortex, and the amplitude of BOLD responses can decrease significantly, bringing to false negatives in the activation map [45]–[47]. This limitation is common to BOLD techniques, therefore both rs-fMRI and tb-fMRI can be impaired. In addition, long-distance interhemispheric connections are vulnerable to injury, therefore rs-fMRI maps may be altered or unavailable [24], [25], [32]. In our study, case 6 with bilateral deficits (left hemiplegia since birth and left hemisphere lesion) had a good tb-fMRI map, but rs-fMRI mapping failed with all methods. Case 3 with glioblastoma multiforme, with edema and severe motor weakness had a satisfactory tb-fMRI map (and ICA map), while aROI and fROI failed to produce convincing results. In another patient with lung metastasis and severe edema (Case 10), ICA failed altogether. While we do not have data on vascular reactivity and baseline perfusion in these patients, the observed variability warns that inter-individual differences in pathophysiology unrelated to neural integrity may be present. Future investigations should combine tb- and rs-fMRI with the study of vascular integrity.

However, while rs-fMRI is more recent and has so far received less attention than tb-fMRI as a potential tool for presurgical planning, there is no hard evidence that the results it provides are less valid than tb-fMRI. In particular, filtering techniques are evolving rapidly and appropriate filtering can substantially increase sensitivity and reduce spurious correlations [36], [48]–[50]. Furthermore, recent work suggests that emerging connectivity-based approaches may attain better statistical power in explaining the neural bases of cognitive and sensorimotor functions than straightforward univariate analyses (e.g. [51]). Future studies will need to assess the relative accuracy of tb-fMRI and rs-fMRI with respect to the ECS gold-standard, and to dissect the effect of preprocessing choices on rs-fMRI maps.

We compared multiple approaches to rs-fMRI data analysis: SBA (aROI, fROI) and ICA. fROI does not represent a proper alternative to tb-fMRI since it implies the availability of a tb-fMRI map, though obtained with the contralateral, healthy hemisoma. Here, fROI was particularly useful as a validity check to verify the adequacy of the anatomical ROIs. fROI and aROI showed samilar overlap sensitivity (α∼0.44) which confirmed that selecting the ROI on the basis of anatomical landmarks is appropriate. By contrast, fROI showed a higher overlap specificity than aROI (β∼0.51 vs β∼0.30), indicating that aROI might include a larger proportion of cortex outside the eloquent regions activated by the task.

ICA and aROI represent the main data analysis approaches: while they gave different results at the level of single cases, on average the overlap sensitivity and specificity values were similar (α∼0.42 and β∼0.32). The ICA sensorimotor component localized well the sensorimotor strip, in particular the hand area, but not the paracentral lobule corresponding to the foot area; this is in line with the established topography of this component as determined on groups of healthy controls [15]. By contrast, the aROI analyses provided activation maps that were more specific to the anatomical area of interest, in particular for the foot area. Therefore, to study the hand area aROI and ICA can be used interchangeably (see Cases 5, 9), whereas to study the foot area, aROI is more indicated (see Cases 12, and 7).

Differences in the results provided by SBA and ICA are not unexpected since SBA and ICA are based on distinct mathematical and physical assumption [15], [28] and differences between aROI and fROI maps plausibly reflect ROI size and thresholding variations. Overall, on the basis of the findings of the present study we suggest to use aROI to localize the foot area, and to use aROI and ICA jointly, whenever it is possible, to study the hand and mouth areas.

As regards the two activation thresholding approaches, on the whole, operator-chosen thresholds and application of the AMPLE criterion produced similar results in terms of correspondence between tb-fMRI and rs-fMRI (Fig. 4). The thresholds chosen for tb-fMRI were numerically similar and strongly correlated (Table 3). For aROI and fROI, moderate-to-strong correlation was observed over cases. For ICA, no significant correlation was found, and manually-chosen thresholds were approximately 30% lower than those given by AMPLE. Notably, for mapping of the foot area with rs-fMRI there was a clear difference between maps generated with operator-chosen and AMPLE thresholds: the latter were too stringent, and this resulted in suppression of activity in the paracentral lobule (Fig. 4b). In addition, AMPLE thresholds applied to ICA maps were more stringent than those chosen by the operators and the resulting ICA sensitivity was reduced compared to the other approach (Table 5 vs. Table 4). Operators apply complex heuristics to converge on a threshold, finding a balance between visibility of activity in the expected anatomical region and presence of spurious clusters in areas distant from the expected region of activation. This process typically involves iteration over several threshold values, and embeds assumptions about where activity should and should not be found based on anatomy. By contrast, AMPLE rests on a simple empirical postulate, i.e. that 50% of the excitation level in the area of expected activation (here, the motor cortex) is generally acceptable as a threshold level. It is different from operation-based determination in that activity outside the motor strip is not considered at all, and that inside the motor strip only the maximum is regarded. As a result, presence of a high maximum can mask activity in other parts of the motor strip. Reassuringly, the two approaches were significantly correlated, and this enabled us to conclude that our correspondence results are not significantly biased by threshold choice. With this, no definite claims are made regarding whether AMPLE or operator-dependent thresholding should be preferred, except that the former is inadequate in the case of foot motor tasks. The two approaches could be combined in future work by utilizing the AMPLE criterion to propose an initial threshold to the operator.

An important issue regards whether rs-fMRI offers some advantages with respect to the other approaches used to activate the sensorimotor network without overt movement, namely motor imagery and passive movement. Motor imagery can be defined as mental simulation of a motor act [52]. Motor imagery and execution have common functional circuits and, importantly, imagining moving various body parts (hand, foot and tongue) activates the precentral gyrus in a somatotopic manner [53]. Notably, the primary motor area is involved in motor imagery [54], [55], but the involvement has been shown to be decreased and inconsistent, and activation appears shifted anteriorly with respect to overt task execution [56]–[58]. However, it is well-known that motor imagery is difficult even for healthy subjects, given the abstraction and attentional load, and there are marked differences also in primary motor cortex between good and bad imagers [59]. Patients may fail a motor imagery task for attentional deficits, cognitive or emotional status. Performance cannot be verified except with real time fMRI, which is not available in all centers.

Passive movement is another approach useful to activate the sensorimotor network, where the affected limb is passively moved by the therapist. The sensorimotor system is activated because the proprioceptive information produced by passive movement not only targets somatosensory but also motor areas [60]. In normal subjects passive movement activates the same areas as execution, although slightly more weakly [58], [61], [62]. In those few patients in whom passive movement has been applied, activation of somatosensory cortex and to a lesser extent of primary motor area was observed, but the activation pattern was very different from that observed in motor execution [58]. Importantly, passive movement is thought to activate the motor system in a rather passive or automatic way, which is only partially affected by reorganization due to brain damage [58]. Therefore, if the aim is to activate the whole motor area as in active tasks, passive movement is inferior to imagery. Accordingly, rs-fMRI can have a role in preoperative clinical settings, in particular in those cases where imagery appears too difficult to be executed.

The present study has some limitations that need to be considered. First, our findings relate to a relatively small sample of patients and require confirmation in larger groups. Second, only ECS points which elicited motor responses on EMG were considered and overall the number of ECS data points was limited. Future studies will need to include positive and negative stimulated sites, investigating the sensitivity and specificity of tb-fMRI and rs-fMRI with respect to intraopertive ECS mapping. This is important because, in spite of its widespread acceptance and reports of significant correlation with ECS, tb-fMRI is not a gold-standard technique, and rigorous assessment of activation detection accuracy can only be performed taking ECS as reference. Third, post-operative performance was assessed with BMRC, a generic scale which does not provide specific information on the limb affected by resection. Hence, we were unable to draw conclusions related to the predictive power of rs-fMRI or tb-fMRI with respect to the outcome. Fourth, in our cohort, only 1 tb-fMRI activation map was unsatisfactory (Case 10, foot motor task). This overall high performance may not be representative and could lead to underestimation of the importance of having an alternative to traditional tb-fMRI, given that the execution of a task can be problematic in presurgical patients due to sensorimotor or cognitive impairment [5], [39]. Fifth, we did not examine systematically the potential effect of head movement during resting and active movement phases. Even though the head displacement values were relatively small, confounding effects cannot be ruled out a priori and future work should characterize in detail the effect of movement on comparisons between tb-fMRI and rs-fMRI, particularly as a function of different preprocessing choices [63]. Sixth, a possible limitation is that lesions were not masked away during anatomical segmentation. In principle, this might have complicated the determination of tissue class contrast distributions, however, the choice was motivated by the fact that lesion boundaries as drawn for volume determination did not include all the surrounding edema when this was present, and anyway lesion masking would not have removed the effect of displacement and distortion of normal landmarks due to mass effect. In practice, thanks to the use of tissue priors, the segmentation procedure implemented in SPM8 is very robust and performs very well even in presence of macroscopical structural damage, such as found in vegetative state patients [36]. Here, the quality of segmentation and subsequent normalization was checked for all cases and the results were deemed adequate (Fig. S1). Seventh, while tb-fMRI general linear-model analyses were performed in native space, and the resulting statistical maps were thereafter normalized, for rs-fMRI the raw volumes were immediately normalized and subsequent analyses (ICA, fROI, aROI) were performed in normalized space. This introduces a potential bias, given that recent work has shown that rs-fMRI analyses performed in native or normalized space do not necessarily yield equivalent results, due to the effect of interpolation [64]. Here, tb-fMRI analyses were performed in native space to avoid any form of spatial manipulation, as the resulting maps were used for presurgical planning and guided ECS. In principle, rs-fMRI analyses could have been conducted in native space, but this would have involved multiple transformations of the seed masks for fROI and aROI, and would have raised issues since the acquisition voxel size was different between the two scanners. At present, it has not be definitely established whether it is preferable to conduct rs-fMRI analyses in native or normalized space, and future work will need to clarify the implications for studies like the present one.

In conclusion, in agreement with previous studies [23], [24], [26], [65] our quantitative evaluation indicates that rs-fMRI can provide valuable information on the anatomy of the sensorimotor system. However, it agrees with tb-fMRI only partially [20]. While this result should not be interpreted as a validation since tb-fMRI is not a gold-standard, it indicates that rs-fMRI can be considered as a supplement to tb-fMRI for presurgical assessment when patients are unable to perform the task or when tb-fMRI fails, but it cannot replace it outright. Until it is formally validated with respect to ECS in a large sample, rs-fMRI should be used with caution. The choice of data analytic approach should be guided by lesion localization, since there are differences in the sensitivity and specificity with respect to activation in the hand, mouth and foot subregions of the motor cortex.

## Supporting Information

Figure S1 **Representative orthogonal sections of normalized task-based fMRI (tb-fMRI) and resting-state fMRI (rs-fMRI) volumes.** The quality of normalization was assessed by two experienced operators and good matching of the brain outline was attained in all cases.
(TIF)
