# Supplementary figures and images for: Preoperative Mapping of the Sensorimotor Cortex: Comparative Assessment of Task-Based and Resting-State fMRI
Source: PLoS One. 2014 Jun 10;9(6):e98860. doi: 10.1371/journal.pone.0098860 (PMC4051640; doi:10.1371/journal.pone.0098860)

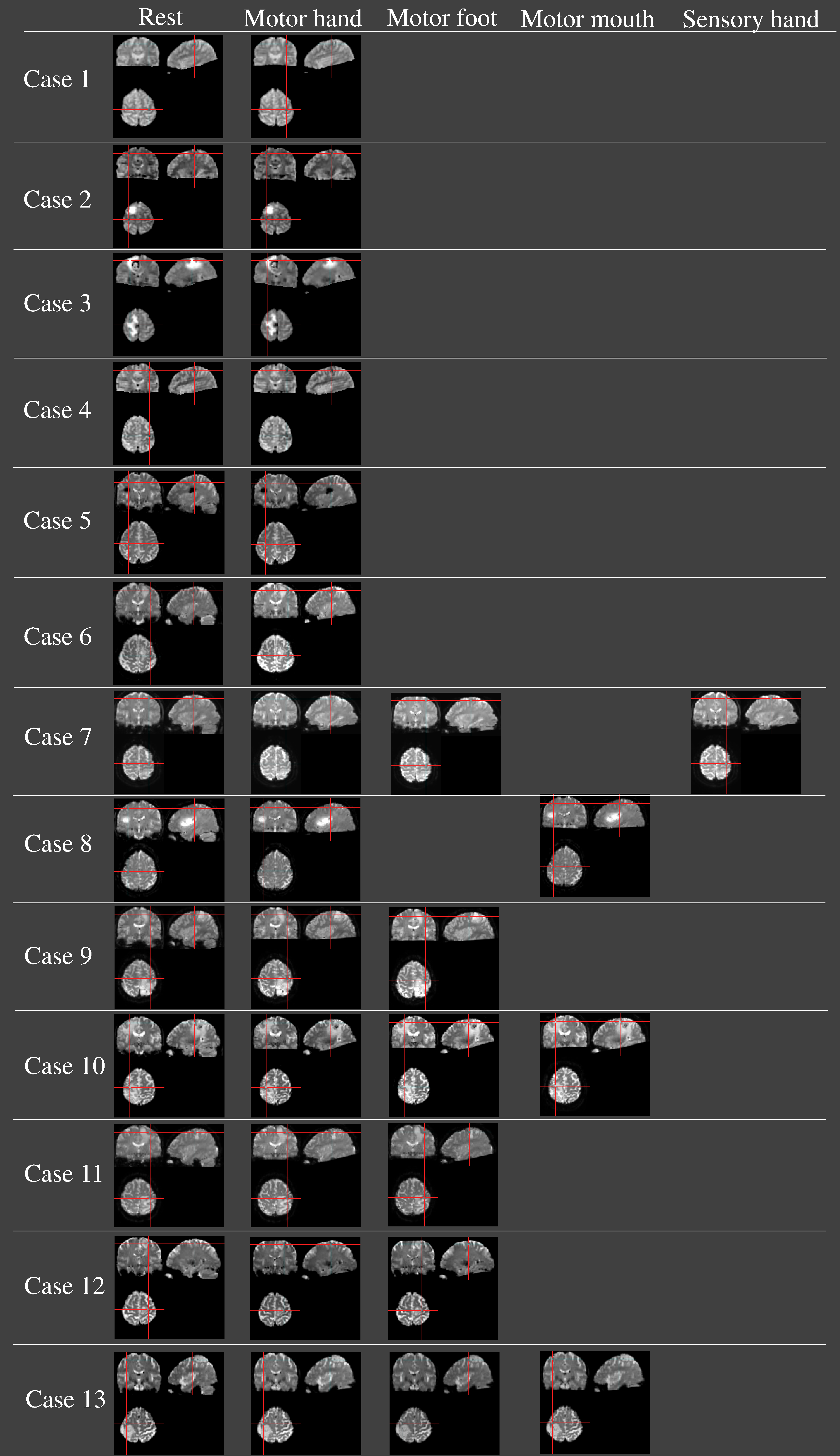

Supplement: Figure S1 — Representative orthogonal sections of normalized task-based fMRI (tb-fMRI) and resting-state fMRI (rs-fMRI) volumes. The quality of normalization was assessed by two experienced operators and good matching of the brain outline was attained in all cases. (TIF) [file pone.0098860.s001.tif]
